# Valerianate of Ammonia for Epilepsy

**Published:** 1858-04

**Authors:** 


### VALERIANATE OF AMMONIA FOR EPILEPSY.

In Salpetriere and the Bicetre at Paris, the following formula has been much used for years:

| | | |
|---|---|---|
| ℞ | Aqua distill., | pts., 95 |
| | Acid valerian, | " 3 |
| | Sub.-carb. ammon., Q. S., ad. neutralis acid adde. | |
| | Ext. alcoholic valerian, | pts., 2. |

Mix. Dose, teaspoonful three times a day.—*Revue Medico-Chirurgicale,*

